# Increasing Soil Organic Carbon but Decoupling of Ecological Attributes After Loss of Dominant Functional Groups in Alpine Meadow

**DOI:** 10.1002/ece3.72134

**Published:** 2025-09-27

**Authors:** Xue Hu, Li Ma, Ruimin Qin, Qiang Zhang, A. Dehaze, Zhen Wang, Zhonghua Zhang, Jingjing Wei, Hongye Su, Shan Li, Zhengchen Shi, Huakun Zhou

**Affiliations:** ^1^ Qinghai Provincial Key Laboratory of Restoration Ecology in Cold Regions, Northwest Institute of Plateau Biology Chinese Academy of Sciences Xining China; ^2^ Academy of Animal and Veterinary Qinghai University Xining China; ^3^ College of Resources and Environment University of Chinese Academy of Sciences Beijing China; ^4^ College of Geographical Science Qinghai Normal University Xining China

**Keywords:** alpine meadow, biodiversity and ecosystem function, dominant species, plant functional group, soil organic carbon, species loss

## Abstract

Due to the sensitivity of alpine meadow ecosystems in the Qinghai‐Tibet Plateau to climate change and human activities, dominant plants are gradually being replaced, potentially triggering ecosystem shifts and affecting soil organic carbon (SOC) sequestration. We conducted a three‐year dominant plant removal experiment to assess impacts on SOC and related ecological attributes. The results showed a significant increase in SOC across all removal treatments, with the greatest increase following the loss of the dominant species. This increase was enhanced by changes in soil moisture and nitrogen, and was also partly attributed to the presence of remaining plant species. On the contrary, SOC accumulation was less responsive to soil moisture and nutrients under the dominant functional group or total removal, indicating that more severe disturbances reduce the supporting factors of SOC. Metal ions also positively influenced SOC following the loss of the dominant plant group. In particular, this study focused on shallow soil layers (0–10 cm), where SOC gains raise concerns about long‐term stability. Correlation analyses and structural equation model revealed that, after the removal of the dominant species, the remaining species maintained the coupling between ecological attributes, preserving ecosystem functioning. However, the removal of dominant functional groups completely disrupted the connections between ecological attributes, reducing the complexity of SOC regulation and key mediating effects. Therefore, prioritizing the restoration of dominant species groups and functional groups is essential for maintaining ecosystem interactions and promoting effective recovery in meadow restoration.

## Graphical abstract

The loss of dominant species or functional groups both leads to an increase in soil organic carbon (SOC), with the loss of dominant species having a stronger effect than that of functional groups. While the removal of dominant species enhances SOC accumulation, it also preserves some coupling among ecosystem attributes, whereas the loss of dominant functional groups completely decouples these attributes. These findings suggest that maintaining dominant species is crucial for sustaining ecosystem interactions and promoting effective recovery.

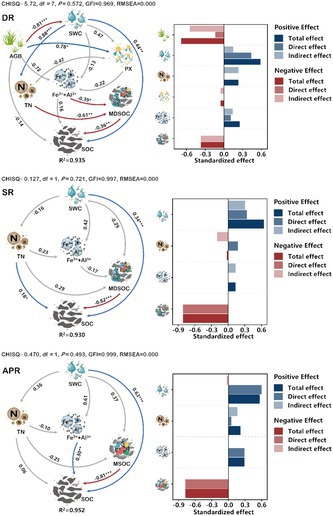

## 1 Introduction

The Qinghai‐Tibet Plateau, often referred to as the “Third Pole of the World” has an average elevation exceeding 4000 m and represents one of the most climate‐sensitive regions on Earth. Recent studies shown that temperatures in this region are rising at approximately 0.4°C per decade—about one to two times faster than the global average (Yao et al. 2022) Compared with other terrestrial ecosystems, the ecological environment of the plateau is more fragile, and intensified human activities have further accelerated its rapid response to climate and environmental changes (Yao et al. 2006). This heightened sensitivity to multiple stressors makes both climate change and human disturbance key drivers of plant functional group succession in the widely distributed alpine meadows across the plateau, with profound consequences for regional ecosystem functioning. Specifically, under the combined influence of climate change (e.g., warming and nitrogen deposition) and anthropogenic activities (e.g., grazing), alpine meadow communities—once dominated by grasses and sedges—are undergoing succession toward more diverse, weed‐dominated communities (Shen et al. 2021; Xu et al. 2024), or even further degrading into communities dominated by low‐biomass annual shrubs and forbs (Brandt et al. 2013; Wu et al. 2024; Liu et al. 2023; Zhang et al. 2022). These shifts in plant functional groups can largely be attributed to the evolutionary adaptation of species to specific environmental conditions (Davis and Shaw 2001), including periodic biological rhythms (Wolf et al. 2017) and differences in resource‐use strategies for light, water, and nutrients (Brantley et al. 2013; Suding et al. 2005; O'Connor et al. 2015; Hautier et al. 2009). However, this succession process, driven by the dual forces of climate change and human activity, not only alters community composition but also fundamentally reshapes the relationship between biodiversity and ecosystem function in alpine meadow systems.

It is well‐established that the diversity and composition of plant communities significantly influence a range of ecosystem processes, including primary productivity, soil nutrient availability (Hooper and Vitousek 1997; Potter et al. 2023), and carbon sequestration (Hooper and Vitousek 1997; Fornara and Tilman 2008). Among these functions, soil carbon sequestration has garnered particular attention, as soils represent the largest carbon reservoir in terrestrial ecosystems—storing approximately two to three times more carbon than the atmosphere. Moreover, carbon dioxide emissions from soil respiration account for over 70% of total terrestrial CO2 emissions (Lal 2023). Consequently, the capacity of soils to sequester carbon is regarded as a key ecosystem function for sustaining the global carbon balance and mitigating climate change. The development and turnover of aboveground vegetation can influence soil organic carbon (SOC) dynamics through multiple pathways. On one hand, changes in litter input during community succession directly affect the quantity and quality of organic matter entering the soil (Prommer et al. 2020), thereby altering both the size and composition of the SOC pool. On the other hand, shifts in vegetation structure can modify soil moisture regimes, nutrient availability, and microbial activity—factors that interactively regulate SOC accumulation and stability (Norton et al. 2012; Xu et al. 2020). In addition to altering soil physical and chemical properties, changes in plant community composition can lead to fluctuations in soil metal element concentrations (Tian et al. 2015). Such changes may further influence SOC stability by mediating the complexation and adsorption of organic matter onto mineral surfaces (Chen et al. 2020; Colombo et al. 2014). Importantly, SOC is not a uniform entity. Its labile fractions—such as dissolved organic carbon (DOC) and microbial biomass carbon (MBC)—are more responsive to subtle environmental changes than more stable carbon forms (Belay‐Tedla et al. 2009; Luo et al. 2015). This high sensitivity not only affects short‐term carbon turnover rates but also determines the long‐term persistence of SOC under changing environmental conditions.

Dominant plant taxa represent a core component of vegetation structure and are crucial drivers of ecosystem functioning. Their changes often accompany substantial shifts in plant community composition and lead to cascading effects on biodiversity, primary productivity, and soil nutrient and moisture dynamics (Liu et al. 2022; Wu et al. 2024; Myers‐Smith et al. 2011). Given that dominant species typically contribute disproportionately to aboveground biomass and resource acquisition, they play a pivotal role in carbon and nutrient cycling by modifying environmental conditions—such as light availability, soil moisture, and temperature—and by regulating the inputs and decomposition of organic matter (Hillebrand et al. 2008; Lohbeck et al. 2016). In particular, dominant plants influence the balance of carbon inputs and outputs through their effects on gross primary productivity, carbon allocation efficiency, and autotrophic as well as rhizosphere respiration (De Deyn et al. 2008). Consequently, their loss may induce dynamic shifts in soil carbon pools. Several meta‐analyses have demonstrated that the loss of dominant species is frequently associated with significant changes in soil organic carbon content (Eldridge et al. 2011; Li et al. 2016). However, the direction and magnitude of this effect remain inconsistent. Some studies report that the disappearance of dominant species can enhance soil organic carbon and total nitrogen accumulation (McKinley and Blair 2008; Throop et al. 2013), while others have reported reductions in carbon stocks (Jackson et al. 2002; Coetsee et al. 2013), or found no significant effects (Lett et al. 2004). Similar uncertainties have been observed in studies investigating the removal of dominant plant functional groups (Melendez Gonzalez et al. 2019; Grau‐Andrés et al. 2020; Chen et al. 2016). These discrepancies may be attributed to variations in environmental context, vegetation types, and the extent to which remaining community members functionally compensate for the loss, all of which warrant further systematic investigation.

The Qinghai‐Tibet Plateau serves as a major carbon sink, with soil carbon accounting for over 90% of the region's total carbon storage (Chen et al. 2022). Even minor fluctuations in the soil carbon pool can significantly influence atmospheric carbon concentrations (Wang et al. 2021). Due to the unique thermal dynamics of the plateau, such changes may further amplify climatic impacts across East Asia and even the Northern Hemisphere through atmospheric circulation (Cheng et al. 1997). Therefore, understanding how ongoing plant community shifts in alpine meadow ecosystems regulate soil organic carbon dynamics is of critical importance. Building on plant removal experiments (Díaz et al. 2003), researchers have examined how the loss of dominant plants—including both dominant species and dominant functional groups—affects soil organic carbon. Evidence suggests that the removal of dominant grass and sedge functional groups leads to a significant decline in soil organic carbon, while microbial biomass carbon exhibits plant group‐specific responses (Chen et al. 2016). Moreover, some studies indicate that dominant species removal produces a delayed effect: although soil organic carbon and its labile fractions may not change significantly in the short term, soil respiration rates exhibit strong altitudinal variation. Li et al. (2018) further demonstrated that even with stable microbial biomass carbon levels, the removal of dominant grass functional groups significantly reduces soil carbon flux. Similarly, Yang et al. (2021) found that excluding dominant grass groups weakens soil decomposition capacity, ultimately decreasing soil organic carbon levels. Although existing studies have offered valuable insights into how plant community succession influences soil organic carbon, two critical scientific questions remain to be addressed. First, the mechanisms through which the removal of dominant plant groups—including both dominant species and dominant functional groups—affects soil organic carbon are still poorly understood, particularly due to limited exploration of the underlying regulatory processes. Second, it remains unclear whether species‐level effects of dominant species or group‐level effects of dominant functional groups play a more decisive role in driving soil organic carbon accumulation.

In summary, the loss of dominant plants in the Qinghai‐Tibet Plateau presents significant uncertainty about its impact on soil organic carbon, and there are considerable gaps in understanding the mechanisms behind changes in soil organic carbon content. Therefore, we plan to conduct an in‐depth investigation within the alpine meadow ecosystems that are widely distributed across the Qinghai‐Tibet Plateau, addressing the uncertainties and gaps identified in previous research. We will focus on two dominant groups in the alpine meadow ecosystem: one is the dominant species, and the other is the dominant functional group. By excluding these two types of species, we will assess whether changes in soil organic carbon will respond to the loss of dominant species and explore the underlying mechanisms. Regarding these research questions, we propose the following hypotheses: (i) the removal of dominant species or dominant functional groups will lead to a significant decrease in soil organic carbon content; (ii) the mechanisms by which the removal of dominant species and dominant functional groups affects soil organic carbon are similar, although there may be differences in the intensity of their impacts.

## 2 Material and Method

### 2.1 Overview of the Study Area and Experimental Settings

**FIGURE 1:**
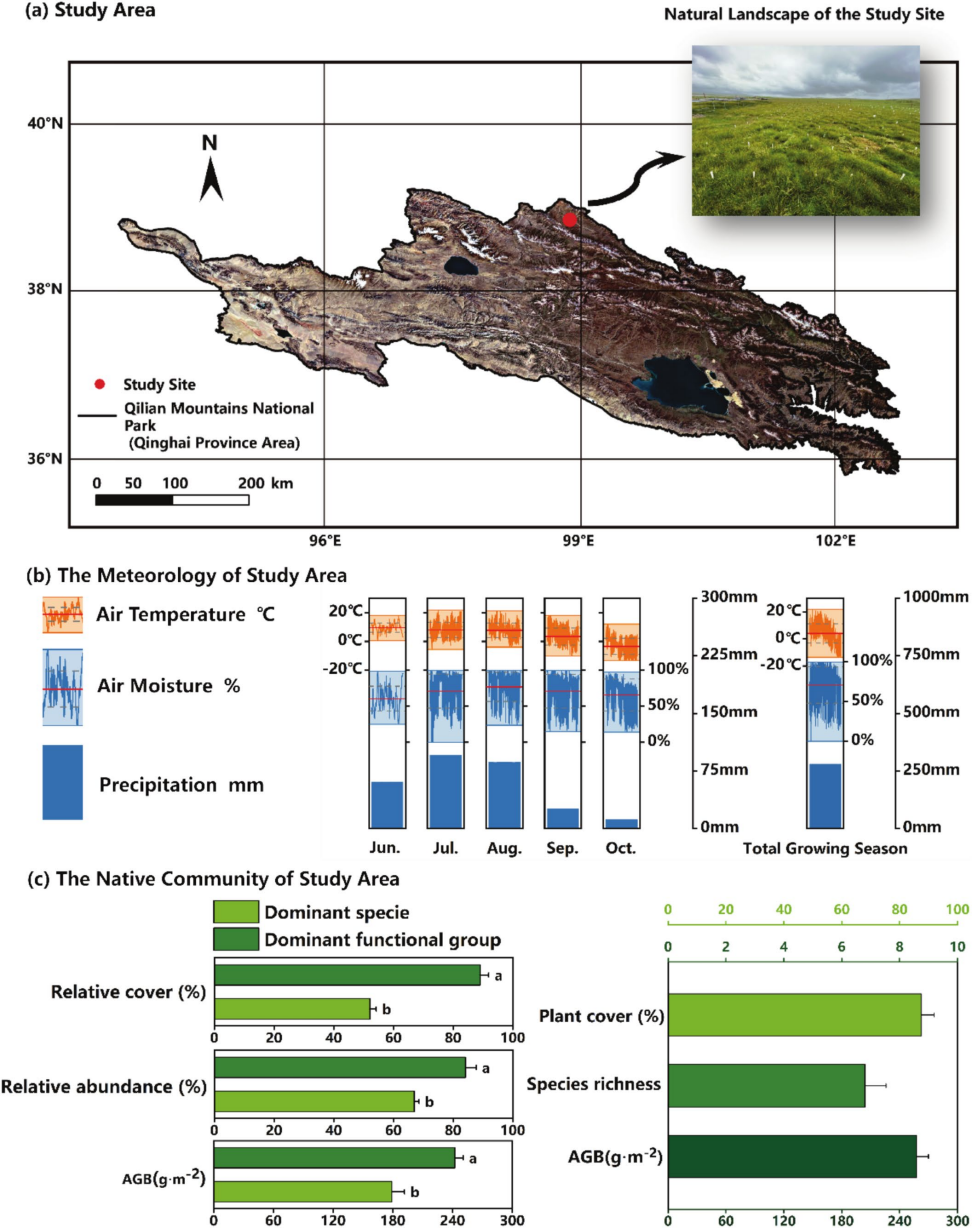
The study area (a) of the Qilian Mountain National Park (Qinghai Province Area) and its meteorological conditions (b) during the growing season and native communities (c).

The study site (38°50′55′ ′′ N, 98°53′23′ E) is located in the northeastern region of the Qilian Mountains National Park (Qinghai Province Area) on the Tibetan Plateau (Figure 1a). Situated at an elevation of approximately 3700 m, the area experiences a high‐altitude plateau climate. The average air temperature during the growing season is around 4.00°C, with an average air humidity of 70.79% and total precipitation of 281.90 mm (Figure 1b). The type of ecosystem in the research area is alpine meadow, with the dominant plant functional group being sedge and the dominant species being *Carex tibetikobresia*, which belongs to this functional group. According to the baseline survey of the native community (Figure 1c), the relative cover of the dominant functional group exceeds 80%, and the cover of the dominant species represents more than half of the total cover of the community. The aboveground biomass of the dominant functional group is close to the total biomass of the community, while the aboveground biomass of the dominant species approaches 70% of the total biomass of the community.

The study used a completely randomized block design across the entire experimental area. The design included an untreated native community (CK) and three removal treatments: removal of dominant species (DR), removal of the dominant functional group (SR), and removal of all plants (APR). Each treatment was replicated five times within four randomized blocks, resulting in a total of 20 plots, each measuring 1.00 m × 0.75 m. To minimize interference between treatments, transition zones were established between the randomized blocks. The removal experiment began in early 2021, with plant growth resumption occurring annually in June. Consequently, plant functional group removal treatments began in June 2021 and continued through September each year. The removal method involved manually cutting plants 2 cm below the soil surface. The removal frequency was one to two times per month to ensure effective treatment. Data collection for this study occurred in 2023, the third year of the experiment, allowing for comparison of ecosystem property changes between treatment areas after 3 years of treatment. The experimental design and procedures were adapted from plant functional group removal experiments conducted at the Haibei Alpine Grassland Ecosystem National Field Science Observation and Research Station (Chen et al. 2016; Wei et al. 2024).

### 2.2 Sample Collection and Measurement

Sampling was conducted from August to late September 2023. Within each plot, a 0.25 m × 0.25 m quadrat was used for sampling. To ensure randomness and minimize edge effects, two sampling points were randomly selected within each quadrat, avoiding edges. All plants within the quadrant were clipped with scissors and placed in paper bags. The aboveground biomass samples from the ground were transported to the laboratory, where they were initially heated at 105°C for 30 min to stop enzyme activity, then dried at 65°C for 48 h until a constant weight was achieved. The samples were weighed using a high‐precision balance to obtain accurate aboveground biomass data. In addition to the collection of aboveground biomass, soil cores with a diameter of 5 cm were taken from the depth of 0–10 cm to include both roots and soil. These samples were immediately sealed and transported to the laboratory for further analysis. In the laboratory, roots were separated from soil samples using a 2 mm stainless steel sieve. The roots were washed under running water several times until no visible soil adhered. The cleaned roots were then dried at 65°C for 48 h, until a constant weight was achieved, and weighed to calculate the belowground biomass. During the root separation process, soil samples were divided into two portions: one portion was air‐dried in a well‐ventilated area for subsequent analysis of the physical and chemical properties of the soil, while the other portion was stored at 4°C for analysis of organic carbon and other sensitive active components.

The soil water content (SWC, g·kg^−1^) was measured using the gravimetric method by drying fresh soil samples at 105°C for 24 h until a constant weight was achieved (Xie et al. 2017). The water loss was calculated based on the initial weight of the soil sample; soil pH was measured using a calibrated pH meter in a soil‐to‐water suspension with a ratio of 2.5: 1. Deionized water was boiled before use to ensure the removal of dissolved gases, which could otherwise affect pH measurements (Haghverdi and Kooch 2019); total nitrogen (TN, g·kg^−1^) was determined using the Kjeldahl digestion method, which involves the conversion of organic nitrogen to ammonium. Ammonium was then quantified using a spectrophotometer at the appropriate wavelength (Barbano et al. 1990); soil peroxidase activity (PX, U·g^−1^) was determined by measuring enzyme‐catalyzed oxidation of organic substrates to quinones, which exhibit a characteristic absorption peak at 430 nm. This method provides insight into the oxidative capacity of soil and its potential to decompose organic matter (Saiya‐Cork et al. 2002); soil organic matter was determined using the potassium dichromate oxidation method. Fe^3+^ and Al^3+^ (mg·kg^−1^) were determined by extracting soil samples with 0.1 M FeCl_3_ or AlCl_3_. After filtration, spectrophotometric analysis was performed. Fe^3+^ was measured using 1,10‐phenanthroline at 595 nm and Al^3+^ was measured using pyrocatechol violet at 430 nm (Sharpley and Smith 1994).

The values of organic matter of the soil were then converted to organic carbon of the soil (SOC, g·kg^−1^) using a standard conversion factor of 0.58 (Krieger 2001); microbial biomass carbon (MBC, mg·kg^−1^) was determined by the fumigation‐extraction method (Brookes et al. 1985). Approximately 4 g of fresh soil were fumigated with chloroform for 24 h, then extracted with 0.5 M K2SO4 using an overhead shaker at room temperature for 1.5 h. The extracts were then filtered through paper filters and soluble organic carbon was analyzed. Dissolved organic carbon (DOC, mg·kg^−1^) was measured by shaking a mixture of soil and deionized water in a 1: 4 soil to water ratio for 1 hour. The mixture was then filtered through 0.45 μm cellulose acetate filters and the DOC content was quantified using a multi N/C 2100 automatic analyzer (Jones and Willett 2006). Furthermore, we calculated the contributions of active fractions of soil organic carbon (SOC) to total SOC. Specifically, the contribution of dissolved organic carbon (DSOC, %) to SOC was determined by dividing the content of dissolved organic carbon by the total SOC content. Similarly, the contribution of microbial biomass carbon (MSOC, %) to SOC was calculated by dividing the microbial biomass carbon content by the total SOC. Furthermore, the combined contribution of microbial biomass carbon and dissolved organic carbon (MDSOC, %) to SOC was derived by dividing the sum of these two fractions by the total SOC content.

### 2.3 Data Analysis

Our data approximately followed a normal distribution and passed the homogeneity of variance test (Figure S1, Table S1). To analyze differences in relative cover, relative abundance, and aboveground biomass between dominant species and dominant functional groups in native plant communities, one‐way analysis of variance (ANOVA) was used. Additionally, ANOVA was used to assess the effects of the three different plant removal treatments on soil organic carbon (SOC) content, the content of labile fractions, and their contribution to SOC, as well as other properties of the ecosystem. Fisher's least significant difference (LSD) and Tukey's post hoc test were applied for multiple comparisons, with significance set at *p* < 0.05. Statistical analyses were performed using IBM SPSS Statistics 26 (IBM, USA), and data visualization was performed with Origin2021 (OriginLab, USA). To explore the correlations among SOC, SOC labile fractions, as well as the contributions of SOC labile fractions to SOC under different plant removal treatments, Spearman correlation analysis was applied. This analysis was also used to investigate correlations among other ecosystem properties. Correlation analyses and visualizations were performed using R (R‐4.3.2) with the *corrplot* package (Wickham 2016), *circlize* package, and *Hmisc* package (Wei and Simko 2017) to generate correlation chord diagrams. Simple linear regression models were used to predict pairwise relationships between each ecosystem property and SOC, SOC labile fractions, or the contributions of SOC labile fractions to SOC under different plant removal treatments. Data analysis and visualization were performed using the *ggplot2* package (Wickham 2016). Both the simple linear regression models and the Spearman correlation analysis between ecosystem properties and SOC dynamics were visualized using the *linkET* package (Harrell Jr and Harrell Jr 2019). A random forest algorithm was applied to further evaluate the contributions of labile carbon fractions and other ecosystem properties to SOC content. The *randomForest* package (Liaw and Wiener 2002) provided the relative importance scores of the predictor variables, while the A3 package was used to assess the significance of the overall model. Visualizations were generated using Origin2021 (OriginLab, USA). Lastly, structural equation models (SEMs) were used to analyze the pathways through which SOC is influenced by different plant removal treatments. SEM analyses were conducted using the *lavaan* package (Rosseel 2012), and model visualizations were produced using the *semPlot* package (Epskamp et al. 2022).

## 3 Result

### 3.1 Comparative Analysis of Soil Organic Carbon Properties Across the Removal Experiment

**FIGURE 2:**
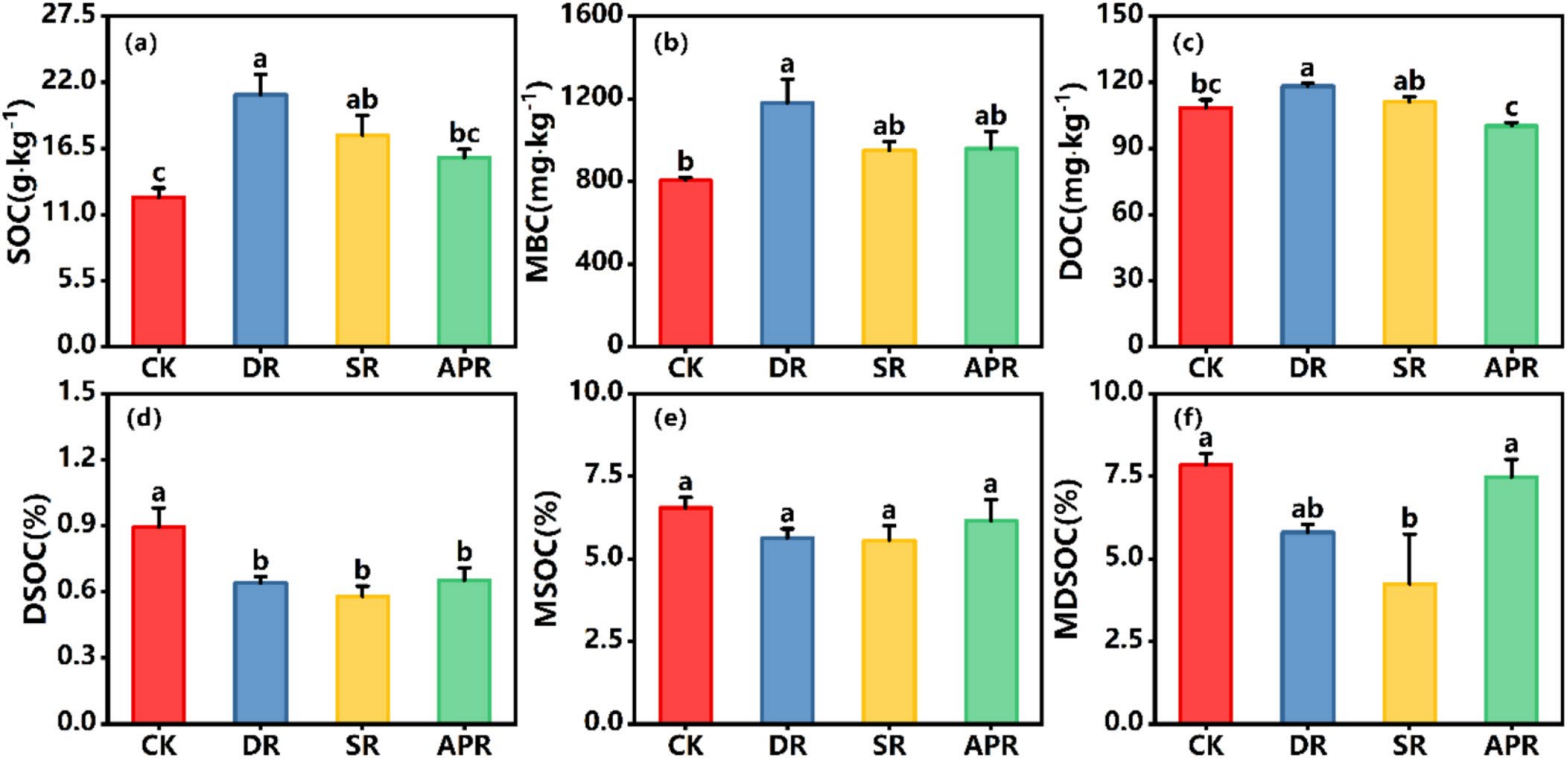
Concentrations of soil organic carbon (SOC)‐, microbial biomass carbon (MBC), dissolved organic carbon (DOC), and the contributions of labile fractions to soil organic carbon after the removal experiment. DSOC, the contribution of DOC to SOC; MSOC, the contribution of MBC to SOC; MDSOC, the contribution of MBC and DOC to SOC. CK, untreated control group; DR, removal of the dominant species; SR, removal of the dominant functional group; APR, removal of all plants. Values followed by a different letter are significantly different within each treatment (*p* < 0.05). (a–f): represent the ANOVA plots for soil organic carbon parameters corresponding to the y‐axis of Figure 2.

After 3 years of removal treatments, the SOC content increased significantly following all removal treatments (Figure 2a). Compared to the native community (CK), the SOC increased by 68.25% in the removal treatment removal of dominant species (DR), representing the largest increase. SOC increased by 41.37% in the removal treatment of the dominant functional group (SR) and by 26.49% in the total removal of vegetation (APR). Furthermore, MBC and DOC showed the greatest increases in the treatment of dominant species removal (Figure 2b,c).

In terms of the contributions of DOC to SOC (DSOC, %), significant reductions were observed after the removal of dominant species and the dominant functional groups, with declines of 28.25% and 35.16% (Figure 2d), respectively. In the total vegetation removal treatment, DSOC decreased by 27.03%. Although the contributions of MBC to SOC (MSOC, %) also decreased in all removal treatments, these reductions were not significant (Figure 2e). In general, the combined contributions of the sum of MBC and DOC to SOC (MDSOC, %) declined in all treatments, with more pronounced decreases in the treatments of removal of dominant species (26.03%) and removal of dominant functional groups (27.04%) (Figure 2f).

### 3.2 Comparative Analysis of Other Ecological Properties in Response to the Experiment of Removal

**FIGURE 3:**
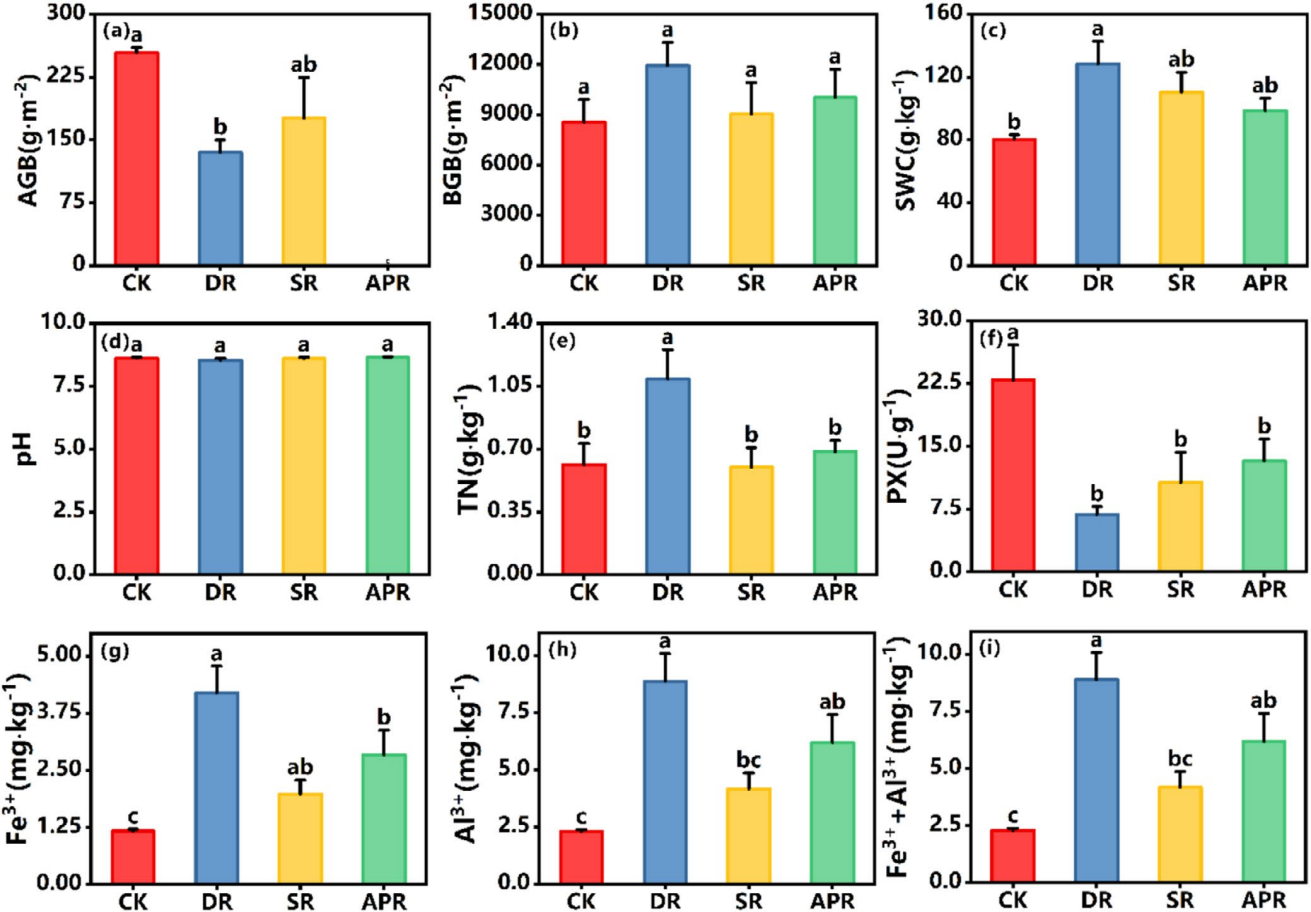
Concentrations of ecological properties following the removal experiment. AGB, aboveground biomass; BGB, belowground biomass; SWC, soil water content; pH, soil pH value; TN, soil total nitrogen content; PX, soil peroxidase activity; Fe^3+^, the concentration of soil ferric ion; Al^3+^, the concentration of soil aluminum ion; Fe^3+^+Al^3+^, the sum of soil ferric and aluminum ion concentration. CK, untreated control group; DR, removal of the dominant species; SR, removal of the dominant functional group; APR, removal of all plants. Values followed by a different letter are significantly different within each treatment (*p* < 0.05). (a–f): represent the ANOVA plots for the ecological properties corresponding to the y‐axis of Figure 3.

After 3 years of removal treatments, other properties of the ecosystem also showed significant changes. AGB decreased significantly in all removal treatments, with the removal of the dominant species showing the greatest reduction at 46.83% (Figure 3a). However, no significant changes were observed in BGB.

The SWC increased across all treatments, with a notable 59.66% increase in the dominant species removal treatment (Figure 3c). The soil pH remained insensitive to any treatment, showing no significant variation. Furthermore, TN increased significantly after the removal of dominant species, with an increase of 77.23% (Figure 3e). PX (U·g ^−1^) decreased significantly in all removal treatments (Figure 3f), with the largest reduction observed in the removal of the dominant functional group (70.07%), followed by the removal of dominant species (53.24%). Regarding soil metal ions Fe^3+^, Al^3+^, and their combined total increased significantly in all removal treatments, with the removal of dominant species showing the greatest increase (Figure 3g–i). In general, ecosystem factors such as biomass aboveground, soil moisture, total nitrogen, and metal ions were most sensitive to the removal of dominant species.

### 3.3 Correlative Analysis of Soil Organic Carbon and Other Ecological Properties in Different Treatments of the Removal Experiment

**FIGURE 4:**
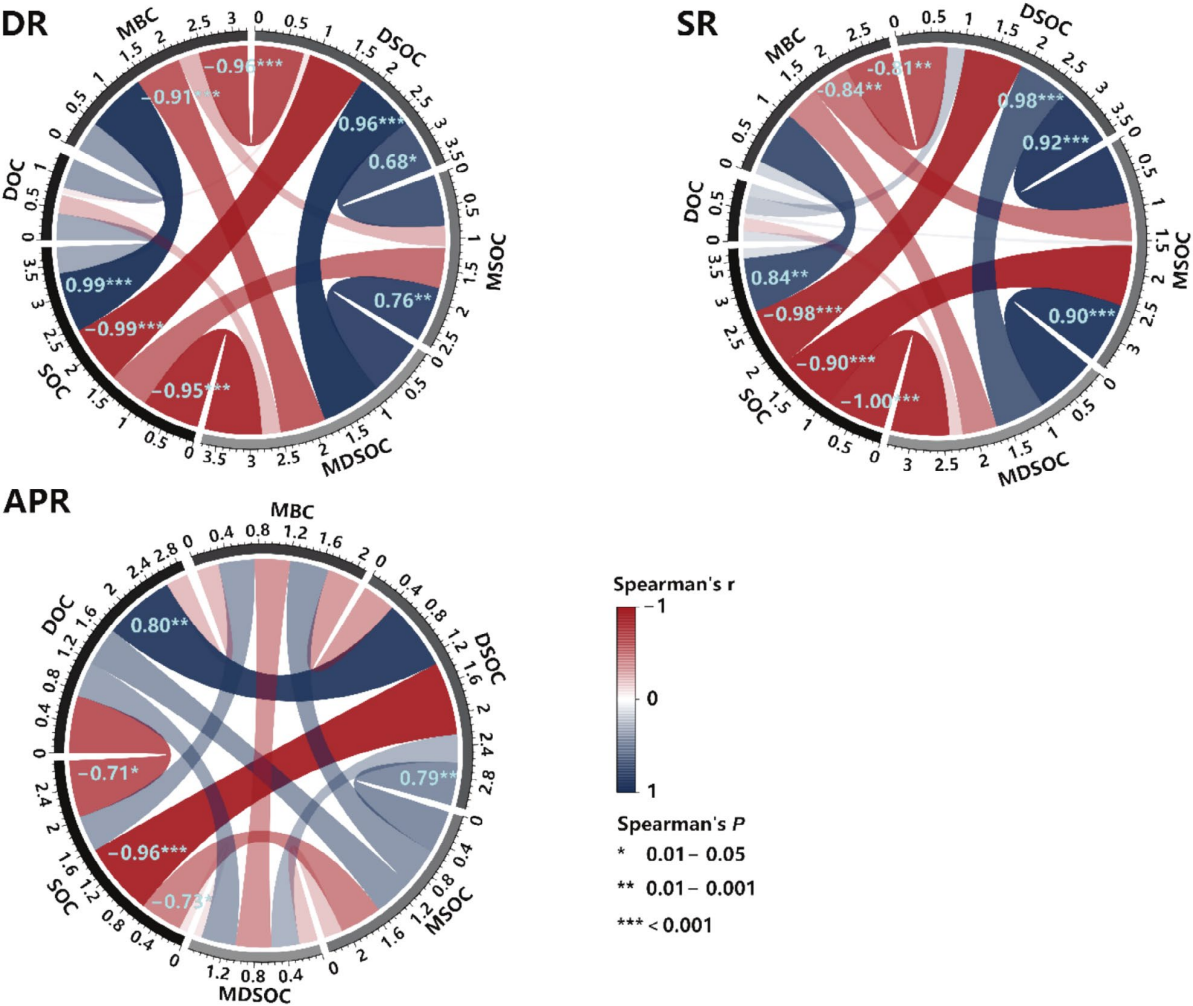
Pairwise Spearman correlation analysis of soil organic carbon properties in removal experiments. In the chord diagram, the width of the chord and the color gradient represent the correlation coefficient, with values annotated on the chords. SOC, soil organic carbon content; MBC, microbial biomass carbon content; DOC, dissolved organic carbon content; DSOC, the contribution of DOC to SOC; MSOC, the contribution of MBC to SOC; MDSOC, the contribution of MBC and DOC to SOC. DR, removal of the dominant species; SR, removal of the dominant functional group; APR, removal of all plants. Significance levels are indicated by asterisks: *p < 0.05, **p < 0.01, ***p < 0.001.

Pairwise correlations of soil organic carbon properties under different plant functional group removal treatments showed distinct patterns. Specifically, after the removal of dominant species (Figure 4, DR), significantly strong positive correlations (*r* > 0.7) were observed between SOC and MBC, DSOC and MSOC, DSOC and MDSOC, as well as MSOC and MDSOC. In contrast, strong negative correlations were detected between SOC and DSOC, SOC and MDSOC, MBC and MDSOC, and MBC and DSOC.

Furthermore, the correlation patterns among the properties of the soil organic carbon were consistent after both the removal of dominant species and dominant functional groups (Figure 4, DR, SR), with differences mainly in the magnitude of the correlation coefficients and significance levels. In comparison, the removal of all plants revealed a unique correlation pattern (Figure 4, APR), where SOC and DOC were significantly negatively correlated, while DOC and DSOC exhibited a significant positive correlation.

**FIGURE 5:**
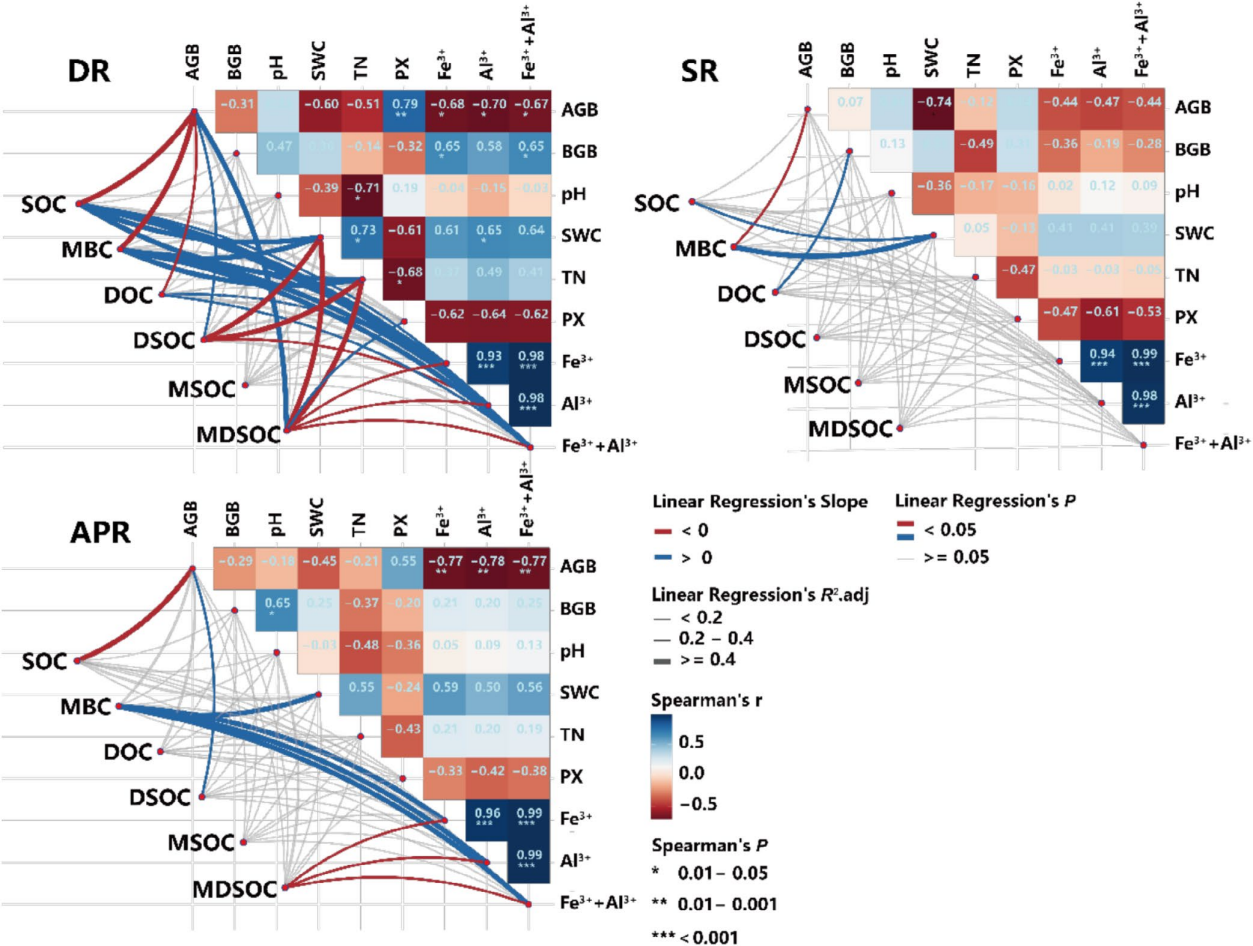
Univariate linear regression analyzes of soil organic carbon properties and other ecological attributes, as well as pairwise correlation analyzes of these attributes, are presented under different removal treatments. In the figures, red and blue lines represent the results of univariate linear regressions between the SOC properties and other ecological attributes. Red lines indicate a significant positive linear relationship (*p* < 0.05), while blue lines indicate a significant negative linear relationship (*p* < 0.05). Gray lines represent non‐significant linear relationships. The thickness of the lines reflects the goodness of fit (*R* ^2^) of the linear regression models. The correlation heat map shows Spearman correlation analyses between ecological attributes, with color gradients representing the Spearman correlation coefficients. SOC, soil organic carbon content; MBC, microbial biomass carbon content; DOC, dissolved organic carbon content; DSOC, the contribution of DOC to SOC; MSOC, the contribution of MBC to SOC; MDSOC, the contribution of MBC and DOC to SOC. AGB, aboveground biomass; BGB, belowground biomass; SWC, soil water content; pH, soil pH value; TN, soil total nitrogen content; PX, soil peroxidase activity; Fe^3+^, the concentration of soil ferric ion; Al^3+^, the concentration of soil aluminum ion; Fe^3+^+Al^3+^, the sum of soil ferric and aluminum ion concentration. DR, removal of the dominant species; SR, removal of the dominant functional group; APR, removal of all plants. Significance levels are indicated by asterisks: *p < 0.05, **p < 0.01, ***p < 0.001.

In the univariate linear regression analysis, the results indicate more complex relationships between soil organic carbon attributes and other ecological variables following the removal of dominant species (Figure 5, DR). Specifically, AGB showed significant negative linear relationship with SOC, MBC, and DOC (*p* < 0.05), whereas it exhibits an opposite linear relationship with DSOC and MSOC. BGB and pH did not have a significant linear effect on any of the attributes of organic carbon attributes (*p* > 0.05). Other soil properties, such as SWC and TN, showed a significant positive influence on SOC and its active components (*p* < 0.05), but the relationship between these properties and the contribution of active components to SOC were negative (*p* < 0.05), indicating that the increase in SOC exceeds the growth of its active components. In contrast, in the treatments involving the removal of dominant functional groups and the complete removal of plants (Figure 5, SR, APR), the univariate linear regression relationships between soil organic carbon attributes and most ecological variables became nonsignificant.

In the Spearman rank correlation analysis, after the removal of dominant species (Figure 5, DR), AGB was significant positive correlation with PX (*p* < 0.01) and significant negative correlation with soil Fe^3+^, Al^3+^ and their combined Fe^3+^+Al^3+^ (*p* < 0.01). BGB was significantly positively correlated with Fe^3+^ and Fe^3+^+Al^3+^(*p* < 0.05). The soil pH was significantly negatively correlated with TN (*p* < 0.05), whereas SWC exhibited significant positive correlations with TN and Al^3+^ (*p* < 0.05). Furthermore, TN was significantly negatively correlated with PX, but positively correlated with iron, aluminum, and iron plus aluminum. On the contrary, after the removal of dominant functional groups (Figure 5, SR) and the complete removal of plants (Figure 5, APR), many of the significant pairwise correlations among ecological attributes were partially lost.

### 3.4 Importance Ranking of Ecological Properties and Soil Organic Carbon Attribute Affecting Soil Organic Carbon in Different Treatments of the Removal Experiment

**FIGURE 6:**
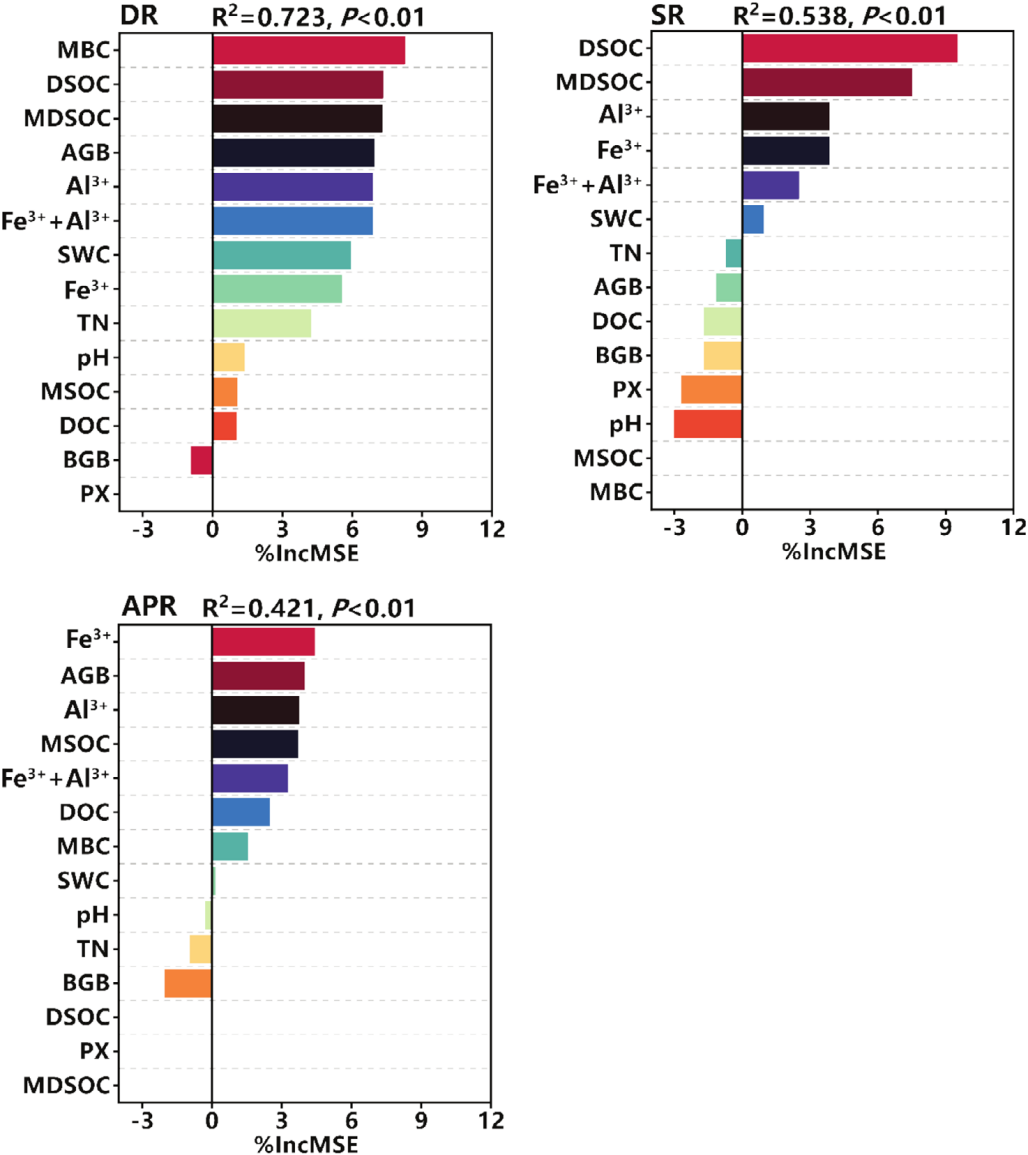
Importance ranking of ecological properties that influence soil organic carbon under different plant functional group removal treatments, assessed using a permutation‐based variable importance method. The percentage increase in mean squared error (% IncMSE) estimates the significance of these predictors, with higher %IncMSE values indicating greater predictor importance. A negative %IncMSE value suggests that the variable contributes little to the model, while missing values indicate that the variable has no measurable effect or contribution, rendering its importance incalculable. MBC, microbial biomass carbon content; DOC, dissolved organic carbon content; DSOC, the contribution of DOC to SOC; MSOC, the contribution of MBC to SOC; MDSOC, the contribution of MBC and DOC to SOC. AGB, aboveground biomass; BGB, belowground biomass; SWC, soil water content; pH, soil pH value; TN, soil total nitrogen content; PX, soil peroxidase activity; Fe^3+^, the concentration of soil ferric ion; Al^3+^, the concentration of soil aluminum ion; Fe^3+^+Al^3+^, the sum of soil ferric and aluminum ion concentration. DR, removal of the dominant species; SR, removal of the dominant functional group; APR, removal of all plants.

In random forest analysis (Figure 6), the %IncMSE values for various ecological properties and certain soil organic carbon attributes showed differences in the variance explained (R^2^) under different plant functional group removal treatments. These variables exhibited greater explanatory power for the variation in SOC in the treatment of dominant species removal. First, the importance ranking of AGB varied across treatments: it ranked within the top four in both the dominant species removal and total plant removal treatments (Figure 6, DR, APR), but droped to eighth place in the functional group removal treatment. On the contrary, soil pH, SWC, and TN had a greater influence on SOC in the dominant species removal treatment compared to other treatments. The influence of soil Fe^3+^, Al^3+^, and their combined sum on SOC remained relatively consistent across the three treatments.

There were significant differences in the predicted soil organic carbon among MBC, DOC, and their contribution to soil organic carbon under different treatments. MDSOC showed similar %IncMSE rankings under the dominant species and functional group removal treatments (Figure 6, DR, SR), whereas MSOC had a more pronounced effect on SOC in the total plant removal treatment (Figure 6, APR). Overall, the impact of MBC and DOC on soil organic carbon varied markedly among all removal treatments. MBC had a greater influence under the dominant species removal treatment (Figure 6, DR), while DOC exerted a stronger effect after the total plant removal treatment (Figure 6, APR).

### 3.5 Structural Equation Modeling of Soil Organic Carbon Response Across Different Treatments of the Removal Experiment

**FIGURE 7:**
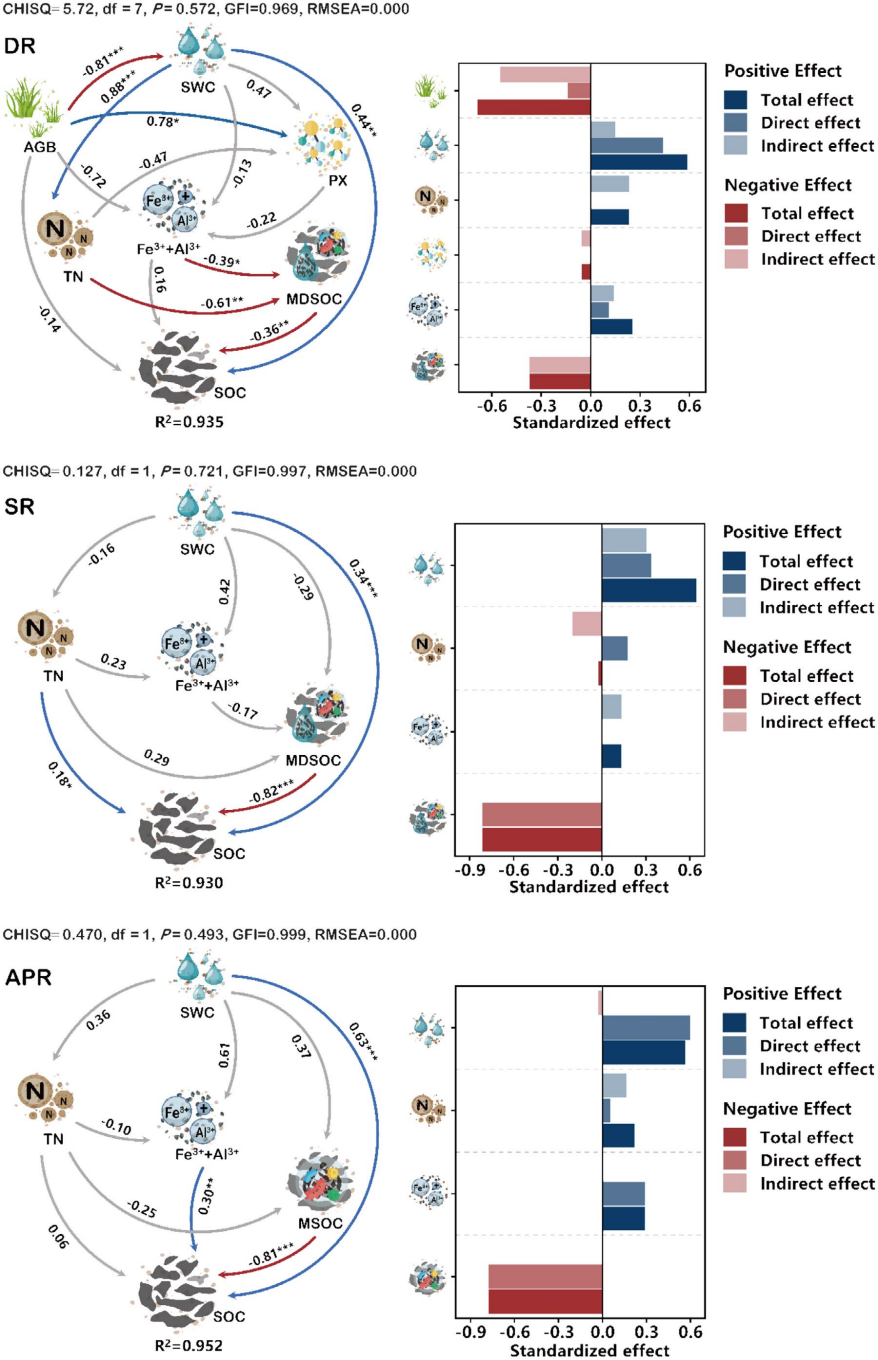
Structural equation models of soil organic carbon content under different plant functional group removal treatments. Arrows represent causal pathways, with red lines indicating significant negative effects, blue lines indicating significant positive effects, and gray lines denoting non‐significant effects. Path coefficients quantify the strength of direct relationships between variables and asterisks indicate significance levels. The bar graphs depict the indirect, direct and total effects of the variables included in the model on the organic carbon content of the soil. CHISQ represents the chi‐square value, df denotes degrees of freedom, P indicates the significance of the model, GFI represents the goodness of fit index, and RMSEA reflects the difference between the theoretical model and the saturated model. SOC, soil organic carbon content; MSOC, the contribution of MBC to SOC; MDSOC, the contribution of MBC and DOC to SOC; AGB, aboveground biomass; SWC, soil water content; TN, soil total nitrogen content; Fe^3+^, the concentration of soil ferric ion; Al^3+^, the concentration of soil aluminum ion; Fe^3+^+Al^3+^, the sum of soil ferric and aluminum ion concentration. DR, removal of the dominant species; SR, removal of the dominant functional group; APR, removal of all plants.

Based on the importance ranking of ecological factors and soil carbon attributes affecting soil organic carbon under different plant functional group removal treatments, structural equation models (SEMs) were constructed to analyze the pathways through which these treatments influence SOC content. The SEMs indicate that the models for the removal of dominant species, the removal of dominant functional groups, and the total removal of plants explained 93.5%, 93.0%, and 95.2% of the variance in SOC, respectively (Figure 7). In the SEM for the removal of dominant species (Figure 7, DR), AGB, MDSOC, and PX exhibited negative effects on SOC. In contrast, other soil attributes included in the SEM generally show positive or minimal negative effects on SOC in all treatments. SWC and MDSOC, MSOC consistently demonstrated strong predictive power for SOC in all three models (Figure 7). Furthermore, the removal of dominant species revealed that more observed variables have significant mediating effects on the explained variables (Figure 7, DR).

## 4 Discussion

### 4.1 Impact on Ecological Properties After the Removal of Dominant Species and Functional Groups

Changes or losses in plant functional groups typically have significant impacts on community biomass (Pan et al. 2016; Xiao et al. 2017), and these biomass changes are associated with the lost functional groups (Pan et al. 2016). In this study, the removal of dominant species and functional groups led to a reduction in aboveground biomass (Figure 3a), indicating that the remaining species may not compensate for the loss of biomass caused by the removal. Dominant species and functional groups are key components of the native Tibetan sedge meadow community, and their loss results in a biomass reduction that is difficult to offset by increasing the biomass of the remaining plants. Similar effects have been observed in other systems, where the loss of dominant species or functional groups exerts a pronounced impact (Smith and Knapp 2003; Wardle and Zackrisson 2005; Longo et al. 2013; Pan et al. 2016). This phenomenon is often explained by the mass ratio hypothesis (Grime 1998). Pan et al. (2016) found that the removal of two dominant plant functional groups led to a significant decline in ecosystem functions, but this decline was avoided when at least one dominant plant functional group remained. This suggests that the characteristics of the dominant groups and their proportions within the community are crucial (Wang and Li 2024). On the contrary, our study did not observe significant changes in belowground biomass after removal treatments (Figure 3b). This lack of significant change may be attributed to limitations in the experimental design: while aboveground biomass was removed, the plant roots remained in the soil, and the cold climate of the study area resulted in slow decomposition rates of dead roots, which could have contributed to the observed stability in belowground biomass (Xu et al. 2015).

In terrestrial ecosystems, the aboveround and belowground subsystems interact to jointly regulate community and ecosystem properties (Wardle et al. 2004). The composition of plant communities is widely recognized as a key factor in controlling the biogeochemical cycles in various ecosystems. This phenomenon is often attributed to the functional traits of the existing dominant plant species and groups (Chapin III et al. 1997; Hooper and Vitousek 1997; Tilman et al. 1997; Wardle and Zackrisson 2005). Our study found that all removal treatments resulted in an increase in soil moisture content (Figure 3c). This may be due to reduced transpiration, decreased moisture absorption by roots, and lower ground cover after vegetation removal, allowing more precipitation to infiltrate the soil (Liu et al. 2024). For example, soil moisture was significantly negatively correlated with aboveground biomass after removal of dominant species, further validating this process. However, removal treatments also led to a reduction in surface plant cover, which in turn exacerbated soil moisture evaporation (Liu et al. 2024). Although the reduction in surface cover due to the removal of dominant species was minor, its impact on soil moisture evaporation was less than that observed with complete plant removal, which still explained the differences in soil moisture content. Similarly, other studies have reported increased soil moisture following removal treatments (Melendez Gonzalez et al. 2019; Andraczek et al. 2023). Additionally, none of the removal treatments had a significant effect on the soil pH (Figure 3d). Although plant removal reduced the number of active roots and root exudates, the impact on soil pH remained minimal, likely due to the strong buffering capacity (McNear Jr. 2013). In other species removal experiments, no significant changes in soil pH were observed (Pan et al. 2016; Zhao et al. 2011; Wardle 1999). Our research also revealed an increase in total nitrogen content in soil after removal of dominant species (Figure 3e), which may be related to the strong control that dominant species have over soil nitrogen levels (Wang et al. 2018). The loss of dominant species leads to nitrogen surplus in the soil, whereas other species in the remaining functional groups promote the activity of nitrogen‐fixing bacteria by altering root exudates, a facilitation that is particularly pronounced in moist soils, resulting in nitrogen accumulation (Koch and Sessitsch 2024). Wardle and Zackrisson (2005) found that the removal of plant functional groups or species increased the amounts of available mineral nitrogen and the ratio of mineral nitrogen to dissolved organic nitrogen. In contrast, the impacts on soil nitrogen from the removal of dominant functional groups and complete removal of plants were similar (Figure 3e). This suggests that whether plants are completely removed or significantly reduced, root functions are largely lost, leading to considerable disturbance in the ecosystem and possibly a new stable state for nitrogen mineralization and nitrification processes (Rastetter et al. 2021). Thus, even with increased soil moisture, the total nitrogen content did not show significant changes. Furthermore, our study found significant decrease in peroxidase activity in all plant functional group removal treatments (Figure 3f), possibly related to the reduced peroxidase content in the soil after functional group removal. After the removal of dominant species, aboveground biomass was significantly positively correlated with soil peroxidase activity (Figure 5, DR), indicating that the removal of aboveground parts resulted in reduced active roots, which in turn decreased the amount of enzymes secreted into the soil (Salem et al. 2022). Other studies have shown that the loss of dominant groups significantly reduced the activity of four carbohydrate hydrolyzing enzymes (e.g., α‐glucosidase, β‐glucosidase, β‐xylosidase, and cellulolytic enzymes) but had no significant impact on phenol oxidase and peroxidase (McLaren et al. 2016). Lastly, our study also found significant increases in Fe^3+^ and Al^3+^ concentrations in soil under different removal treatments (Figure 3g–i). This may be related to reduced plant uptake, increased soil erosion (Lv et al. 2023), changes in soil chemical properties (Munyaneza et al. 2024), and the decomposition of plant litter resulting in higher levels of iron and aluminum ions. Plants typically release organic compounds to chelate trivalent iron ions (Fe^3+^), facilitating their absorption (Colombo et al. 2014). When plants are removed, the reduction in active roots and root exudates leads to decreased chelation, resulting in the accumulation of Fe^3+^ in the soil (Colombo et al. 2014). Furthermore, the removal of plants can alter the soil microbial community, and some microbes may potentially promote the oxidation of divalent iron ions (Fe^2+^) to trivalent iron ions (Fe^3+^), further increasing the concentration of Fe^3+^ (Gao et al. 2024). In particular, the increase in Fe^3+^ and Al^3+^ concentrations in the soil after the removal of dominant species was greater than that observed with the removal of dominant functional groups or complete plant removal.

However, other studies have shown that the loss of dominant plant groups or other functional groups did not significantly affect soil properties (including pH, moisture, and nutrients) (Marshall et al. 2011; Chen et al. 2016). Additionally, Naeem et al. (1994) found significant differences in responses to biodiversity loss across various ecosystem processes. Thus, the findings of this study may reflect the increased sensitivity of the Tibetan Plateau to climate change, amplifying the response of alpine ecosystems to species loss. Furthermore, the relatively short duration of the removal experiment (only 3 years) may have limited the observation of significant changes in ecosystem properties, as long‐term removal experiments may reveal compensatory effects within communities that mask substantial shifts in ecosystem attributes.

### 4.2 Impact on Soil Organic Carbon Attributes After the Removal of Dominant Species and Functional Groups

Changes in aboveground vegetation can affect the ecosystem's ability to absorb atmospheric CO_2_, thereby altering the quantity and quality of organic matter input into the soil (Prommer et al. 2020; Thakur et al. 2024), ultimately impacting soil organic carbon stocks. Typically, the loss of dominant species leads to reduced biomass and, consequently, less organic matter entering the soil, resulting in lower soil organic carbon levels (Dlamini et al. 2014; Kuzyakov et al. 2016). However, in this study, we found that the removal of dominant species, functional groups, and all plants increased both the content of soil organic carbon and microbial biomass carbon, which is contrary to our initial hypothesis. In particular, there was no significant change in the underground biomass of the community (Figure 3b), suggesting that the contribution of decomposing roots to soil organic carbon may be limited. In the correlation analysis after the removal of dominant species, we observed significant negative correlation between aboveground biomass and soil organic carbon, as well as its active components, while soil moisture and total nitrogen content showed significant positive correlations with soil organic carbon and its active components (Figure 4, DR). The structural equation model for the removal of dominant species (Figure 7, DR) indicated that the reduction in aboveground biomass directly increased soil moisture, which subsequently promoted the accumulation of soil organic carbon. Furthermore, the increase in total nitrogen indirectly led to decrease in the contribution of active organic carbon to total soil organic carbon, thus also facilitating its accumulation. This forces us to reconsider the reasons for the increase in the carbon pool in the soil. Specifically, the removal of dominant species led to increased soil moisture and elevated total nitrogen levels. Soil moisture acts as a solvent for nutrients and a carrier for ecological processes; during the gradual disappearance of dominant groups in alpine meadows, the reduction in aboveground vegetation decreased the ability of the grass canopy to intercept precipitation, further increasing soil moisture. Furthermore, microbes are highly sensitive to changes in soil moisture (Tecon and Or 2017; Hao et al. 2025), and the loss of aboveground vegetation has enhanced microbial competition for soil nutrients (Cui et al. 2019), thereby improving microbial decomposition of the remaining organic matter and leading to an increase in soil organic carbon. Microbial biomass carbon, as an active component of soil organic carbon, can quickly respond to environmental changes (Luo et al. 2015; Belay‐Tedla et al. 2009), and the observed increase in microbial biomass partially validates this process (Figure 2b). Increased soil nitrogen availability after the loss of dominant species leads microbes to selectively utilize nitrogen in nitrogen‐rich environments while neglecting carbon sources, thus lowering decomposition rates (Hobbie et al. 2012; Zhou et al. 2014; Zhao et al. 2023), which can also result in organic carbon accumulation. Additionally, structural equation models for the removal of dominant functional groups and all plants indicated similarly (Figure 7, SR, APR) that increased soil moisture promotes the accumulation of soil organic carbon. When dominant functional groups or all plants are removed, plant roots no longer retain soil moisture or contribute organic matter, leading to decreased soil moisture and insufficient supply of nutrients. Under low nutrient and low humidity conditions, the efficiency of microbial carbon use efficiency declines (Manzoni et al. 2012), resulting in microbes allocating more carbon to maintenance and survival rather than growth and reproduction, which in turn releases CO_2_ during decomposition rather than fixing it into stable organic carbon. This may explain why the increase in soil organic carbon is less pronounced after the loss of dominant functional groups and all plants compared to the loss of dominant species. Root exudates are also an important source of dissolved organic carbon (DOC) (Guggenberger and Zech 1994; Thurman 2012). Following the removal of dominant species and functional groups, the content of dissolved organic carbon increased, but decreased after the removal of all plants (Figure 1c). Specifically, when all plants are removed, the pathway for organic matter secretion from roots to the soil disappears, resulting in the inability to compensate for the dissolved organic carbon consumed by soil microbes. Therefore, we predict that the content of soil organic carbon and its active components will continue to decline after the removal of all plants. Additionally, the decrease in the contribution of active organic carbon to soil organic carbon can promote an increase in soil organic carbon content (Figure 7; Shi et al. 2023), indicating that the carbon generated by microbial activity is more likely to be fixed or converted into a more stable organic carbon pool rather than existing directly as active organic carbon (DOC and MBC). Furthermore, the increase in trivalent iron and aluminum ions can provide mineral protection to the formed soil organic carbon (Chen et al. 2020; Colombo et al. 2014) as observed in our structural equation models. The model for the removal of dominant functional groups also showed a non‐significant positive pathway through iron and aluminum ions that regulate the soil organic carbon (Figure 7).

However, it is important to note that the shallow soil layer serves as a crucial transformation zone for organic carbon input from plants, making its organic carbon more susceptible to external influences (Wang et al. 1999; Dungait et al. 2012). Although the loss of dominant groups has promoted soil organic carbon accumulation in the short term, this increase is primarily concentrated in shallow soil (0–10 cm). This layer may be subject to long‐term risks from factors such as oxygen exposure (Wang et al. 2013), active microbial activity (Brangarí et al. 2022), and moisture fluctuations (Overby et al. 2002; Brangarí et al. 2022), all of which can elevate the risk of organic carbon loss and its release into the atmosphere.

When predicting early ecological impacts in changing environments, it is crucial to consider the interactions between biotic and abiotic components, as ecosystems are highly sensitive to certain disturbances, such as severe degradation. Yang et al. (2023) characterized the coupling of ecological properties using correlations between ecosystem attributes, revealing significant decoupling among them. In our study, the loss of dominant functional groups and the complete removal of plants produced similar results. After 3 years of species loss experiments, we found that the negative impacts on the native ecosystem were more pronounced with the removal of dominant functional groups compared to the removal of dominant species. First, in our correlation analysis (Figure 5), the relationship between vegetation biomass and soil properties was more significant after removing dominant species than after complete removal of plants, both in terms of the number of correlations and the strength of the correlation coefficients. Second, in the structural equation models (Figure 7), the model for the removal of dominant species showed that soil organic carbon content was influenced by multiple factors and pathways, while the model for the removal of dominant functional groups was simpler. This indicates that after the removal of dominant species, the ecological properties still maintained a certain level of coupling. In contrast, the removal of dominant functional groups and all plants resulted in a nearly complete decoupling of ecosystem properties. Furthermore, the correlation between vegetation biomass and soil properties after the removal of dominant functional groups was more similar to that observed after the complete removal of plants, suggesting that the remaining functional groups had a weaker capacity to maintain coupling between soil properties. This evidence highlights that dominant functional groups significantly determine the ability of soil ecological processes to resist decoupling, underscoring their important role in maintaining ecosystem stability. Therefore, grassland restoration management strategies should consider improving the rebuilding of dominant groups to restore severely disturbed grasslands, thus strengthening interactions among ecosystem properties for better recovery outcomes. In a broader context, human activities exacerbate ecosystem degradation by increasing industrial input, and climate change influences human migration patterns in various ways (Ullah et al. 2023; Han et al. 2024). Similarly, the evolution of plant communities in the alpine meadows of the Qinghai‐Tibet Plateau—such as the decoupling of ecosystem attributes—further underscores the importance of effectively managing environmental and ecological changes to maintain ecological stability.

## 5 Conclusion

In general, the loss of dominant species in the alpine meadows of the Qinghai‐Tibet Plateau has led to an increase in soil organic carbon (SOC) levels, but the pathways promoting SOC accumulation vary depending on the specific dominant group lost. Following the removal of dominant species, the remaining species played an important role in improving soil properties and promoting SOC accumulation, while maintaining the coupling of ecosystem attributes. On the contrary, the loss of dominant functional groups resulted in a significant reduction in the correlation between soil properties, both in terms of the number and strength of significant relationships, and the SOC regulation pathways became simplified, lacking mediating effects. This evidence suggests a decoupling of ecosystem attributes. Although the increase in SOC content due to the loss of dominant groups is noteworthy, this increase occurs primarily in shallow soil layers, which can increase the risk of SOC pool loss. Furthermore, the presence of dominant groups is crucial for the interactions among ecosystem attributes. Therefore, in future grassland management practices, it is essential to emphasize the role of dominant groups in maintaining ecosystem integrity.

However, this study is limited to non‐random plant diversity loss caused by experimental or artificial manipulation, whereas natural vegetation succession and species turnover in real‐world ecosystems are far more complex. Additionally, although this study focuses on the alpine ecosystems of the Qinghai‐Tibet Plateau, similar shifts in vegetation composition are occurring across high‐latitude ecosystems globally. These changes may have profound consequences for soil carbon storage and present a potential challenge for climate change mitigation. Therefore, future research should aim to investigate the mechanisms of vegetation‐driven SOC dynamics under natural community succession and expand the geographical scope to include high‐latitude or other climate‐sensitive regions.

## Author Contributions

**Xue Hu:** data curation (lead), formal analysis (lead), methodology (lead), writing – original draft (lead). **Li Ma:** conceptualization (equal), investigation (equal). **Ruimin Qin:** investigation (equal), software (equal). **Qiang Zhang:** investigation (equal), methodology (equal). **A. Dehaze:** investigation (equal), software (equal). **Zhen Wang:** methodology (equal), writing – review and editing (equal). **Zhonghua Zhang:** conceptualization (equal). **Jingjing Wei:** methodology (equal). **Hongye Su:** software (equal). **Shan Li:** software (equal). **Zhengchen Shi:** investigation (equal). **Huakun Zhou:** project administration (lead).

## Conflicts of Interest

The authors declare no conflicts of interest.

## Supporting information


**Figure S1:** QQ plots of normality for different analytical indicators.
**Table S1:** Levene's test for homogeneity of variance.

## Data Availability

Our research data is openly available and can be accessed through the following https://github.com/hx13327690616/my‐text‐datafiles/blob/69d2a26cded9ccc091c31798a57a6e876be1a974/data1. We have made the data publicly available to promote transparency and facilitate further research.
