# Supplementary material for: Increasing Soil Organic Carbon but Decoupling of Ecological Attributes After Loss of Dominant Functional Groups in Alpine Meadow
Source: Ecol Evol. 2025 Sep 27;15(10):e72134. doi: 10.1002/ece3.72134 (PMC12475975; doi:10.1002/ece3.72134)
Supplement: Supplementary file 1 — Figure S1: QQ plots of normality for different analytical indicators. Table S1: Levene's test for homogeneity of variance. [file ECE3-15-e72134-s001.docx]

# **Appendix A**

The prerequisites for conducting ANOVA are that the data conform to a normal distribution and pass the test for homogeneity of variances. Therefore, we used the *“ggpubr”* package in R 4.3.2 to generate QQ plots for all indicators in our dataset to assess data normality. For data that met the normality assumption, we then performed Levene's Test for homogeneity of variances using the *“car”* and *“dplyr”* packages in R 4.3.2.


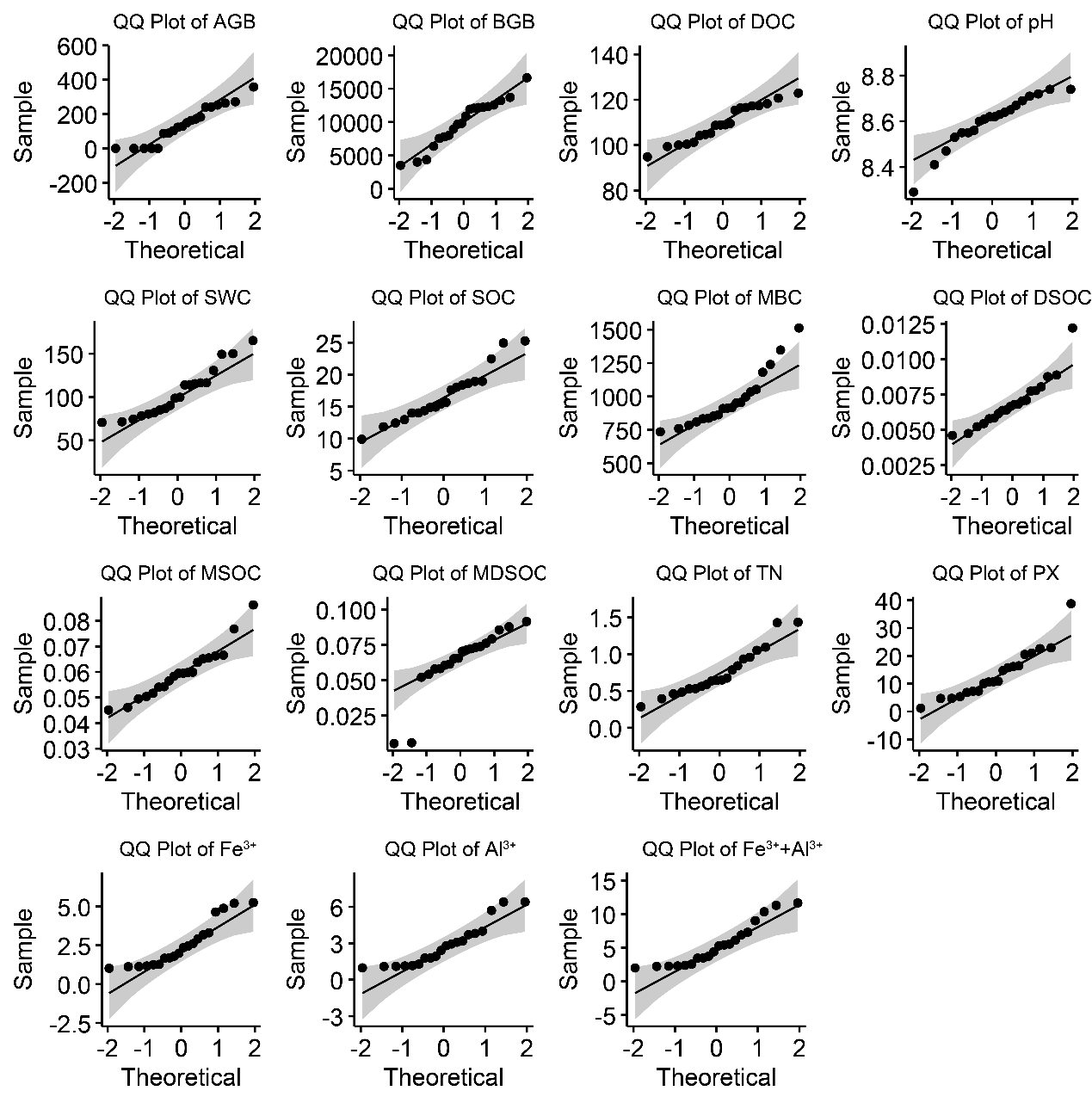


Figure S1 QQ plots of normality for different analytical indicators. AGB, aboveground biomass; BGB, belowground biomass; SWC, soil water content; pH, soil pH value; TN, total nitrogen content; PX, soil peroxidase activity; SOC, soil organic carbon content; MBC, microbial biomass carbon content; DOC, dissolved organic carbon content; DSOC, the contribution of DOC to SOC; MSOC, the contribution of MBC to SOC; MDSOC, the contribution of MBC and DOC to SOC; Fe^3+^, the concentration of soil ferric ion; Al³⁺, the concentration of soil aluminum ion; Fe³⁺+Al³⁺, the sum of soil ferric and aluminum ion concentration.

Table S1 Levene's Test for Homogeneity of Variance

| Indicator | Df_Group | Df_Residual | F_value | p_value |
| --- | --- | --- | --- | --- |
| AGB | 3 | 16 | 2.7231 | 0.0788 |
| BGB | 3 | 16 | 0.2303 | 0.874 |
| DOC | 3 | 16 | 0.9243 | 0.4515 |
| pH | 3 | 16 | 0.473 | 0.7054 |
| SWC | 3 | 16 | 1.675 | 0.2123 |
| SOC | 3 | 16 | 1.1295 | 0.3668 |
| MBC | 3 | 16 | 2.9714 | 0.0631 |
| DSOC | 3 | 16 | 0.6668 | 0.5846 |
| MSOC | 3 | 16 | 0.406 | 0.7508 |
| MDSOC | 3 | 16 | 2.3045 | 0.1158 |
| TN | 3 | 16 | 0.905 | 0.4604 |
| PX | 3 | 16 | 1.1051 | 0.376 |
| Fe^3+^ | 3 | 16 | 1.7489 | 0.1974 |
| Al^3+^ | 3 | 16 | 1.627 | 0.2226 |
| Fe^3+^+Al^3+^ | 3 | 16 | 2.6987 | 0.0805 |

Note: AGB, aboveground biomass; BGB, belowground biomass; SWC, soil water content; pH, soil pH value; TN, total nitrogen content; PX, soil peroxidase activity; SOC, soil organic carbon content; MBC, microbial biomass carbon content; DOC, dissolved organic carbon content; DSOC, the contribution of DOC to SOC; MSOC, the contribution of MBC to SOC; MDSOC, the contribution of MBC and DOC to SOC; Fe^3+^, the concentration of soil ferric ion; Al³⁺, the concentration of soil aluminum ion; Fe³⁺+Al³⁺, the sum of soil ferric and aluminum ion concentration.
